# Late somatic sequelae after treatment of childhood cancer in Slovenia

**DOI:** 10.1186/1756-0500-5-254

**Published:** 2012-05-24

**Authors:** Nuša Erman, Ljupčo Todorovski, Berta Jereb

**Affiliations:** 1 Faculty of Administration, University of Ljubljana, Gosarjeva 5, SI-1000, Ljubljana, Slovenia; 2 Institute of Oncology Ljubljana, Zaloška cesta 2, SI-1000, Ljubljana, Slovenia

## Abstract

**Background:**

This is a long-term follow-up clinical study of adolescents and adults, survivors of childhood cancer. We evaluate and analyze the late somatic sequelae of childhood cancer treatment. Many such studies are susceptible to a strong selection bias, i.e., they employ a limited non-systematic sample of patients, based on a clinical hospital that provided the cancer treatment or performed the follow-up. To address the issue of selection bias, we perform here an analysis of late sequelae on a systematic database of the entire population of the children treated for cancer in Slovenia. Due to the specifics of cancer treatment procedures in Slovenia, they have all been treated and followed-up in the same clinic.

**Methods:**

The data are based on the centralized registry of cancer patients in Slovenia and present a controlled and homogeneous collection. Late sequelae are evaluated following a modified CTCAE, i.e., the National Cancer Institute’s Common Terminology Criteria for Adverse Events version 3.0. We use survival analysis method to estimate the incidence of and risk for late sequelae, where the time variable is measured in years from the diagnosis date, while we follow the event of incidence of late sequelae scored other than none. Survival analysis is performed using KaplanMeier estimator and Cox regression model.

**Results:**

The incidence of mild, moderate, or severe late sequelae of childhood cancer treatment significantly decreased from 75% in the group of patients diagnosed before 1975 to 55% for those diagnosed after 1995. The Cox regression analysis of the risk factors for the incidence of late sequelae identifies three significant factors: treatment modalities, age at diagnosis, and primary diagnosis.

**Conclusions:**

The change of treatment modalities in terms of replacement of surgery and radiotherapy with chemotherapy is the main reason for the decrease of the incidence and the risk for late sequelae of childhood cancer treatment; treatment modalities including surgery significantly increase the risk ratio of late sequelae, while those based on chemotherapy only significantly decreases the risk. Risk of late sequelae increases with the diagnosis age: younger children are more susceptible to late effects of treatment. Finally, primary diagnosis significantly influences the risk for late sequelae, but mostly due to the dependency of the treatment modality on the primary diagnosis.

## Background

In Slovenia and in the rest of Europe as well as the USA, the mortality rate of chlidren with cancer is declining despite the increasing incidence rate. Survival of children treated for cancer has significantly improved after 1970 with the introduction of chemotherapy. Since 1990, however, the rate of increase might have slowed for several reasons: on one hand, new ways of treatment have been introduced and on the other hand, the mortality due to late effects of treatment and secondary tumors have emerged [1-7]. With the increasing number of childhood cancer survivors, late sequelae and quality of life have become a major concern. Approximately 50% of children cured of cancer will display one or more delayed somatic sequelae, and about one third will have severe and life threatening complications. There is therefore a general agreement for the necessity of a long-term follow-up of adolescents and adults, survivors of childhood cancer [8-12].

Childhood cancer survivors included in many long-term follow-up clinical studies do not appropriately represent the entire survivor population for different reasons. One is that not all the patients included in the follow-up respond to the questionnaires or show-up for the regular clinical checks. Another reason is selection bias; studies often address a non-systematic sample of patients based on a clinical hospital that provided the treatment or performed the follow-up. Integrating groups of patients from different follow-up programs or clinics in a more systematic sample of patients is often prohibitive, since there are many different approaches to the evaluation of the late sequelae and quality of life. Finally, that there are still many open issues related to the childhood cancer survivors, such as definitions of the terms cure, health, or quality of life. Even the excellent studies on large populations of patients and based on high-quality questionnaires may be susceptible to selection bias [13-15].

In this paper, we address the problem of selection bias by performing a study of late sequelae on the entire population of all registered children treated for cancer in Slovenia. Namely, the registration of cancer patients in Slovenia is obligatory and centralized within the Cancer Registry of Slovenia, established in 1950. Treatment of children with cancer is also centralized at the Children’s Hospital in Ljubljana. After treatment, all patients are being followed at the same single center for at least five years or until they are 18 years old, and later they are followed regularly at a single institution, the outpatient Clinic for Late Effects at the Institute of Oncology, Ljubljana. The follow-up program that includes a specifically designed questionnaire for recording data has been established in 1986 [16]. Slovene Ministry of Science has been financing the program for “Late effects of childhood cancer treatment” since 1993. Survivors included in the program have been evaluated and analyzed as to their late somatic sequelae including neurological deficits [17], endocrine function deficits [18], renal function damage [19], secondary tumors [20,21], cardiac damage [22], and psychosocial consequences [23].

With increased knowledge about late effects of cancer treatment, treatment policies have been appropriately changed in order to decrease toxic sequelae [24]. For example, Hodgkin's disease treatment with MOPP (i.e., Nitrogen Mustard, Oncovin, procarbazine, and prednisone), known for causing male sterility, has been replaced by other chemotherapy (CHT) combinations, while the doses of radiation have been diminished. In nephroblastoma, radiotherapy (RT) has been replaced in the great majority of patients by chemotherapy. The toxic dose of antracyclines has been avoided in newer combinations of CHT and RT doses have been diminished in many cases of soft tissue sarcoma as well. In more general terms, while in the first decade until 1970 the great majority of children were treated with surgery and RT, in the 1970-ies to 1980-ies for the majority of patients a combination of RT and CHT has been added to surgery. After 1985, RT was often replaced by CHT. Because RT has been shown as the major risk for late sequelae, it is reasonable to expect a decrease of late sequelae in our patients treated after 1985.

The aim of this study is to find out whether the risk for late somatic sequelae has declined with the treatment changes described above. Our study includes all children diagnosed and treated with cancer in Slovenia between 1957 and 2005. The continuous effort on systematic and unified follow-up of all childhood cancer survivors in Slovenia allows us to perform a systematic study and analyze the impact of the treatment advances and changes to the quality of their life.

## Methods

### Patients

The study is based on a systematic database of 2005 children aged 16 years or less registered at the Cancer Registry of Slovenia from 1957 to 2005. Twenty-two have been excluded from the study: nine were lost from follow-up immediately after treatment and for thirteen patients the revision of the primary diagnosis proved that the tumors were not malignant.

Of the remaining 1983 patients, 1130 have not been followed for different reasons: 850 died prior to the first follow-up evaluation, 247 are not eligible for evaluation yet (they are either younger than 18 years or have been treated less than five years ago), 14 refused follow-up, and 19 have been lost from follow-up. Table 1 summarizes the data about the remaining 853 patients in the study group, including information on gender, age at diagnosis, diagnosis, secondary tumor incidence, therapy, and current status by the diagnosis period.

**Table 1:** Patient database summary

		**Diagnosis period**					
	**Patients**	**1957-1975**	**1976-1980**	**1981-1985**	**1986-1990**	**1991-1995**	**1996-2005**
Female	376	39	46	54	69	63	105
Male	477	56	69	89	103	75	85
Age at diagnosis up to 5 years	248	38	38	59	69	39	5
From 5 to 10 years	216	28	44	41	39	37	27
From 10 to 18 years	389	29	33	43	64	62	158
Leukemia	174	13	23	32	41	35	30
CNS	144	24	18	20	30	23	29
HD	123	11	16	24	24	17	31
NHL	87	6	8	17	22	14	20
Renal tumors	45	9	9	11	10	5	1
Neuroblastoma	25	4	3	5	6	6	1
Rhabdomyosarcoma	34	3	6	5	7	9	4
Malignant bone tumors	51	4	4	9	7	8	19
Soft-tissue sarcomas	65	9	13	11	11	9	12
Other carcinomas	70	8	8	3	5	9	37
Other malignant neoplasms	35	4	7	6	9	3	6
No secondary tumor	736	69	92	117	154	128	176
Secondary tumor	117	26	23	26	18	10	14
Surgery	489	66	71	76	100	73	103
Radio therapy	515	74	76	105	110	61	89
Chemo therapy	590	27	82	113	137	101	130
Alive	808	81	106	136	166	135	184
Dead	45	14	9	7	6	3	6
All	853	95	115	143	172	138	190

Summary of the data collected from the Cancer Registry of Slovenia for the 853 patients in the study group by diagnosis period. The figures report number of patients by gender, age at diagnosis, diagnosis, the incidence of secondary tumor, therapy and life status.

The childhood cancer survivors are followed until they are 18 years old at the Center for treatment of children with cancer at the Children’s Hospital. After that, physicians that were involved in the primary treatment collaborate in the follow-up for late effects performed at the next-door Oncological Institute. The children and their parents are well informed about the necessity of a lifetime follow-up by the psychologist that joins the team during treatment. Every childhood cancer survivor is formally invited to a follow-up evaluation and receives a booklet with the necessary information [25]. The great majority of them, 853 in total, accept our recommendation of a lifetime follow-up, resulting in data from not only newly treated patients but also those treated decades ago. The first follow-up evaluation is performed at least five years after the first treatment and at the age of 18 years.

### Follow-up evaluation and scoring

For each follow-up evaluation, the examining physician follows a questionnaire and registers all findings using his clinical judgment. During the 24 years starting from 1986, two clinical investigators have regularly followed the patients in close mutual cooperation, working simultaneously at the outpatient clinic. For scoring of late somatic sequelae, we used a modification of The National Cancer Institutes Common Terminology Criteria for Adverse Events version 3.0 (CTCAE), which includes the classification of organs, the evaluation of the degree of late effects, and the scoring system. The scoring system used in this study, presented in Table 2, is closely related to the scoring system used earlier in a report from the childhood cancer study [26]. However, due to the limited number of patients, some score groups have been merged together following a clinical examination of the patient at the outpatient clinic. Patients with no clinical symptoms and pathological laboratory test that does not require medical treatment have been classified not to have late sequelae (score 0 in Table 2). Not all patients, namely, were subjected to a laboratory test and therefore, the results could not represent the whole series. Patients where deviations from normal findings could not be expected were exluded from laboratory testing, e.g., a patient with only surgical treated glioma of the optic nerve was not subjected to evaluation of endocrine organs, pulmonary function, or kidney function. He was blind on one eye and his sequaelae is classified as moderate (score 2 in Table 2). For a more detailed description of the scoring system, check the Additional file 1.

**Table 2:** Late-sequelae scoring system

** Score**	**Description**
0 (none)	No effect that can be related to cancer treatment or an effect so mild that it requires no regular medication or other healthcare intervention and does not interfere with normal life.
1 (mild)	Mild effects related to cancer treatment that are entirely or almost entirely controlled by medicine and/or other healthcare intervention and do not lead to anything more than a minor alternation in lifestyle.
2 (moderate)	Moderate adverse effects related to cancer treatment that require continued use of medicine and/or other healthcare intervention. The patient remains able to lead an independent existence although some modifications in activity and in style of leaving are necessary.
3 (severe)	Severe adverse effects. The patient’s life is considerably affected by the adverse effects related to cancer treatment with a considerable and continued restriction of activities. Inpatient care and major surgery might have been required.
4 (death)	Death related to the cancer and/or cancer treatment.

The system for scoring late somatic sequelae of childhood cancer and treatment used in our study adapted from [26].

**Figure 1:**
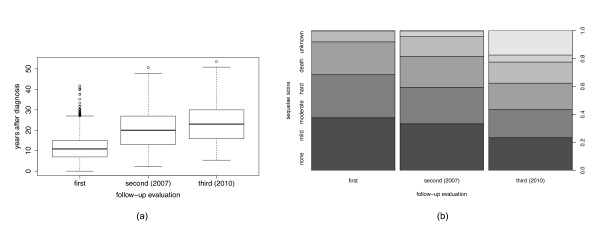
**Late sequelae evaluations.** The period between the diagnosis and the follow-up evaluation (left-hand side, **a)** and the percentage of patients with different evaluation scores from Table 2 (right-hand side, **b)**, for the first follow-up evaluation after treatment, the second one performed in December [3], and the third one in December 2010.

We applied the scoring system on three follow-up evaluations of each patient: at the first evaluation of the late effects, at the follow-up performed in December 2007, and at the last follow-up prior to this study performed in December 2010. The graph in Figure 1(b) shows that the incidence of somatic late sequelae increases with the time period after treatment. At the fist evaluation, almost 40% of patients have no sequelae, while at the third evaluation in 2010, the percentage of patients with no sequelae drops to slightly more than 20%. The box plot graphs in Figure 1(a) show that the time period from the treatment to the first follow-up evaluation varies a great deal for different patients; it can be performed soon after treatment (the bottom whisker of the left-most boxplot), which happens in the cases where secondary tumor is diagnosed soon after the treatment of the primary one, or more than 40 years after treatment (the top whisker of the left-most boxplot). For example, a two-year old child treated for Wilm's tumor in 1990, had his first evaluation at 18 years, that is 16 years after treatment (in 2006), while a 15-year old patient with NHL, treated the same year, had his first evaluation 5 years after treatment (in 1995).

To study sequelae at a fixed time period (of five and ten years) after treatment, we “extrapolated” the sequelae score. For the patients with no sequelae, 10 or more years after treatment, we assume that no sequelae have been observed before the first evaluation date and also five or ten years after treatment. On the other hand, those who had severe sequelae in early years after treatment (at the date of diagnosis of the first secondary cancer) were assumed to have severe sequelae also in the future. Those with moderate score, if there was no change between the first and second evaluation, could be considered to have the same score five years after treatment. Using this method, we were able to “extrapolate” the sequelae five and ten years after treatment for 832 and 785 patients, respectively. Table 3 summarizes the data about the change of the “extrapolated” late somatic sequelae scores for patients in the study group five and ten years after treatment through different diagnosis periods.

**Table 3:** Late-sequelae scores

**Years after treatment**	**Sequelae score**	**Patients, %**	**Diagnosis period**					
			**1957-1975**	**1976-1980**	**1981-1985**	**1986-1990**	**1991-1995**	**1996-2005**
Five	0 (none)	318, 37.3%	23, 24.2%	29, 25.2%	42, 29.4%	72, 41.9%	68, 49.3%	84, 44.2%
	1 (mild)	299, 35.1%	40, 42.1%	45, 39.1%	55, 38.5%	73, 42.4%	40, 29.0%	46, 24.2%
	2 (moderate)	157, 18.4%	18, 18.9%	28, 24.3%	31, 21.7%	18, 10.5%	19, 13.8%	43, 22.6%
	3 (severe)	50,5.9%	6,6.3%	7,6.1%	9,6.3%	8,4.7%	9,6.5%	11, 5.8%
	4 (death)	8,0.9%	0,0.0%	0,0.0%	1,0.7%	1,0.6%	1,0.7%	5,2.6%
	Unknown	21,2.5%	8,8.4%	6,5.2%	5,3.5%	0,0.0%	1,0.7%	1,0.5%
Ten	0 (none)	271, 31.8%	21, 22.1%	26, 22.6%	37, 25.9%	67, 39.0%	66, 47.8%	54, 28.4%
	1 (mild)	241, 28.3%	25, 26.3%	33, 28.7%	48, 33.6%	63, 36.6%	38, 27.5%	34, 17.9%
	2 (moderate)	186, 21.8%	33, 34.7%	33, 28.7%	37, 25.9%	28, 16.3%	21, 15.2%	34, 17.9%
	3 (severe)	71,8.3%	8,8.4%	15, 13.0%	16, 11.2%	10, 5.8%	10, 7.2%	12, 6.3%
	4 (death)	16,1.9%	1,1.1%	2,1.7%	1,0.7%	4,2.3%	2,1.4%	6,3.2%
	Unknown	68,8.0%	7,7.4%	6,5.2%	4,2.8%	0,0.0%	1,0.7%	50, 26.3%

Late somatic sequelae scores for the 853 patients in the study group five and ten years after treatment by diagnosis period. The figures report number and percentage of patients with different late-sequelae scores.

### Data analysis methods

**Figure 2:**
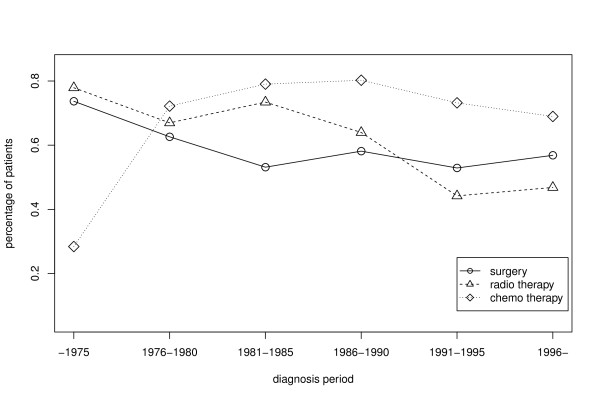
**Treatments through diagnosis periods.** Percentages of patients that received surgery, radiotherapy, and chemotherapy in different diagnosis periods.

Table 3 depicts a trend of increasing percentage of patients with no sequelae five or ten years after treatment through diagnosis periods, from 24% of patients diagnosed and treated before 1975 to almost 50% of those diagnosed between 1991 and 1996. This decrease in the incidence of late sequelae coincides with the dynamic of treatment modalities depicted in Figure 2. Before 1975, most of the patients were treated with a combination of surgery and radiotherapy, while after 1975 chemotherapy treatment prevails. Our hypothesis is that the dynamic change of the therapy combinations through the time has a positive impact on the late sequelae.

To test this central hypothesis, we use survival analysis methodology that allows analysis of the survival time of patients. In general, survival analysis can tackle the occurrence of any particular event of interest and not only death. In our study, the event of interest is late sequelae; more specifically, we follow the occurrence of late sequelae. We measure the time to the occurrence of late sequelae in years from date of the diagnosis and treatment of the primary tumor. We censor data about the patients that do not reach late sequelae evaluation other than none at the time of their last follow-up evaluation. To measure the probability of late sequelae occurrence five and ten years after treatment, we use Kaplan-Meier estimator [27]. We also analyze the hazard function that models the late-sequelae risk, i.e., the probability that the patient has late sequelae other than none at a certain time after treatment. Using a Cox’s proportional hazards model, commonly referred to as Cox regression [28], we analyze the impact of demographic, diagnosis, and treatment factors on the late-sequelae risk (hazard ratio). All tests of significant differences are two-sided with a significance level of 95%. The statistical analysis is performed using the R software environment for statistical computing [29].

## Results

### Late-sequelae occurrence and risk through diagnosis periods

**Figure 3:**
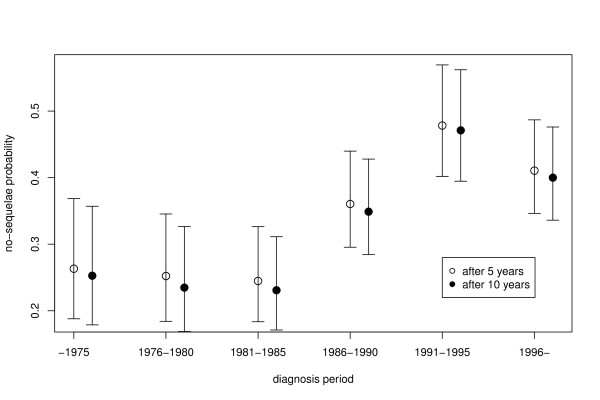
**Late sequelae five and ten years after treatment.** The Kaplan-Meier estimates of the probabilities (and the confidence intervals thereof) that a patient will not have any signs of late sequelae (i.e., the sequelae score will be 0 or none) five (white circles) and ten years (black circles) after treatment. The graph depicts the dynamic change of the probabilities through various diagnosis periods.

Figure 3 depicts the dynamic change of the probability that a patient does not have late sequelae five and ten years after treatment through different diagnosis periods. The figure shows the expected small and consistent decrease of the probability when the observation period extends from five to ten years. More importantly, the figure also shows increase of the probability for no sequelae through time. For the patients treated in the initial periods from 1957 to 1985, the probability is almost constant and below 30%. After 1986, we observe a steep increase of the no-sequelae probability up to the maximal value of almost 50% in the period from 1991 to 1995.

Since the observed change of probability seems to be correlated to the change of therapy modalities depicted in Figure 2, we continue our analysis with the analysis of the treatment modalities on the occurrence of late sequelae. Note that one patient with neuroblastoma diagnosed at birth has not received any treatment; we excluded that patient data from the further analysis.

### Influence of therapy on the late-sequelae risk

**Figure 4:**
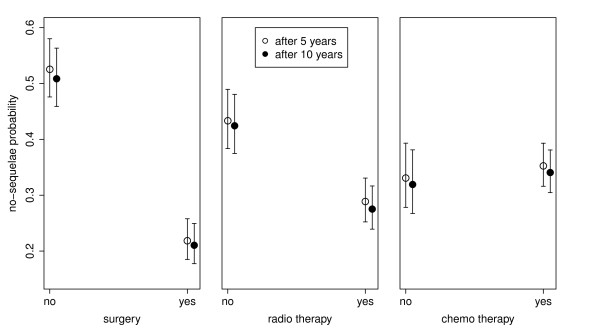
**Influence of treatment on late sequelae incidence.** The influence of individual therapeutic treatment on the Kaplan-Meier estimates of the probabilities (and the confidence intervals thereof) that a patient does not have late sequelae, i.e., the sequelae score of 0 or none is observed, five years (white circles) and ten years (black circles) after treatment.

Figure 4 represents the Kaplan-Meier estimate of probability that a patient has no late effects five and ten after treatment. The figure shows that surgery and RT are much more offensive in terms of late sequelae than CHT. The probability of no late effects is slightly above 50% for patients that have not been subjected to surgery and it significantly drops to slightly above 20% for those that have been treated with surgery. Similarly, the same probability significantly drops from about 45% for patients that have not been treated with RT to slightly below 30% for patients subjected to RT. On the other hand, the probability of no late effects is about 35% for both patients with or without CHT.

These results confirm our hypothesis that the improvements of therapy combinations have a strong impact on the decrease of late-sequelae incidence. However, to test the importance of therapy combination relative to other factors, we perform a multivariate Cox regression analysis.

### Relative importance of late-sequelae risk factors

Table 4 reports the results of the multivariate analysis in terms of the relative impact of gender, age at diagnosis, diagnosis period, therapy, and primary diagnosis on the late sequelae hazard ratio. As expected, gender does not influence the risk for late sequelae: there is only insignificant (p = 0.06) difference between the risk in the reference group of females (hazard ratio of 1.0) and males (hazard ratio of 0.85). On the other hand, age at diagnosis has a significant impact on the late-sequelae risk: it decreases with increasing age. For the group of patients diagnosed at the age between 5 and 10 years as well as 10 and 16 years, late-sequelae risk is significantly lower as compared to the reference group of patients diagnosed when less than 5 years old (hazard ratios of 0.65 and 0.40 as compared to the reference value of 1, with both p-values lower than 0.01).

**Table 4:** Late-sequelae hazard ratios for different risk factors

**Risk factor**	**HR**	**CI**	**P**
Gender: Female (reference group)	1.00		
Male	0.85	0.71–1.00	0.06
Age: from 0 to 5 years (reference group)	1.00		
From 5 to 10 years	0.65	0.51–0.84	<0.01
From 10 to 16 years	0.40	0.31–0.52	<0.01
Diagnosis period: 1957-1975 (reference group)	1.00		
1976-1980	1.15	0.83–1.59	0.41
1981-1985	1.22	0.89–1.68	0.21
1986-1990	1.02	0.74–1.41	0.89
1991-1995	0.90	0.63–1.28	0.56
1996-2005	1.14	0.81–1.61	0.45
Treatment: Surgery only (S, reference group)	1.00		
Radiotherapy only (RT)	1.91	1.06–3.47	0.03
Chemotherapy only (CHT)	0.96	0.53–1.74	0.88
Surgery and Radiotherapy (S_RT)	3.25	2.31–4.57	<0.01
Surgery and Chemotherapy (S_CHT)	2.49	1.76–3.54	<0.01
Radio and Chemotherapy (RT_CHT)	2.29	1.54–3.40	<0.01
Surgery, Radio and Chemotherapy (S_RT_CHT)	3.80	2.71–5.32	<0.01
Primary diagnosis: Leukemia (reference group)	1.00		
NHL	1.82	1.21–2.74	<0.01
Neuroblastoma	2.27	1.23–4.20	0.01
Other malignant neoplasms	2.93	1.69–5.06	<0.01
Rhabdomyosarcoma	2.97	1.79–4.93	<0.01
HD	3.09	2.14–4.45	<0.01
CNS	3.60	2.33–5.55	<0.01
Soft-tissue sarcomas	4.00	2.51–6.38	<0.01
Malignant bone tumors	4.54	2.81–7.31	<0.01
Other carcinomas	4.62	2.73–7.84	<0.01
Renal tumors	6.02	3.71–9.76	<0.01

Cox regression estimates of the late sequelae hazard ratios (HR) by gender, age at diagnosis, diagnosis period, therapy, and primary diagnosis, 95% confidence intervals (CI) thereof, and the p-values for the test of the significant difference from the indicated reference groups.

Results on the impact of treatment reconfirm our findings about the significant impact of the treatment modality on the late-sequelae risk. Following the results, we can identify three categories of treatment modalities. The first category of patients treated with S only and CHT only has low late-sequelae risk (the hazard ratio of 1 and 0.96). Furthermore, the second category has medium risk and includes patients treated with RT only (hazard ratio of 1.91), a combination of RT and CHT (hazard ratio of 2.29), or a combination of surgery and CHT (hazard ratio of 2.49). Finally, patients in the third category with high risk for late sequelae include those treated with the combinations of surgery and RT (hazard ratio of 3.25) or surgery, RT, and CHT (hazard ratio of 3.80).

Another significant risk factor turns out to be primary diagnosis. The reference group of patients diagnosed with leukemia has a significantly lower late-sequelae risk compared to any other primary diagnosis. However, a deeper analysis of the dependence of treatment on diagnosis, presented in Table 5, shows that this is mostly due to the treatment modalities applied in the context of the primary diagnosis. Majority of Leukemia patients (97%) were treated with CHT only (a treatment modality with the lowest late-sequelae risk) or a combination of RT and CHT (a treatment modality with medium late-sequelae risk). Similarly, in the group of patients diagnosed with NHL (hazard ratio of 1.82), 67% of the patients were treated following these two modalities. On the other hand, none of the patients diagnosed with renal tumors (the group with the highest hazard ratio of 6.02) received CHT only or a combination of RT and CHT and 47% received the most invasive combination of surgery, RT, and CHT. From the results presented in Table 5, we can conclude that the impact of the primary diagnosis on the late-sequelae can be mostly explained through its influence on the selection of the treatment modality.

**Table 5:** Treatment modality by primary diagnosis

**Treatment**							
Primary diagnosis	S	RT	CHT	S_RT	S_CHT	RT_CHT	S_RT_CHT
Leukemia	0.6%	1.1%	27.0%	0.6%	0.0%	70.1%	0.6%
NHL	2.3%	3.4%	25.3%	3.4%	16.1%	41.4%	8.0%
Neuroblastoma	37.5%	4.2%	0.0%	8.3%	25.0%	4.2%	20.8%
Other malignant neoplasms	40.0%	2.9%	2.9%	20.0%	25.7%	0.0%	8.6%
Rhabdomyosarcoma	2.9%	0.0%	0.0%	0.0%	20.6%	29.4%	47.1%
HD	0.0%	5.7%	4.1%	8.9%	1.6%	59.3%	20.3%
CNS	31.9%	3.5%	0.0%	35.4%	2.1%	2.8%	24.3%
Soft-tissue sarcomas	24.6%	0.0%	0.0%	10.8%	50.8%	1.5%	12.3%
Malignant bone tumors	3.9%	0.0%	0.0%	7.8%	60.8%	13.7%	13.7%
Other carcinomas	60.0%	2.9%	0.0%	18.6%	5.7%	4.3%	8.6%
Renal tumors	2.2%	0.0%	0.0%	11.1%	40.0%	0.0%	46.7%

The influence of the primary diagnosis on the treatment modality.

## Discussion

The survival analysis of childhood cancer patients has been used in a number of other studies, including a study of the same data in Slovenia [1]. In the context of late sequelae, survival analysis has been recently used in [2], where CTCAE has been applied to evaluate late somatic sequelae of 519 childhood cancer survivors with late sequelae other than none. The results on the incidence of late sequelae are similar to the results of our study; most of the late sequelae other than none are mild or moderate. The study also identifies four risk factors for severe sequelae of childhood cancer treatment: minority race, diagnosis of other tumor, older age at diagnosis, and a history of stem cell transplant. The comparison with our analysis of risk factors for any late sequelae other than none, reveals a single common factor of age. However, in our study, results show that higher age at diagnosis lowers the risk for late sequelae.

Other studies of childhood cancer survival and sequelae confirm our results on significant improvements gained through different diagnosis periods especially after 1980, while no decrease in the late mortality rate by treatment period (the periods being 1960–1970 and 1971–1984) has been identified in [30].

## Conclusions

Although there is general agreement on the need for lifelong follow-up of childhood cancer survivors, the transfer of former patients from pediatric to satisfactory adult cancer care is not easy to accomplish [31-33] and the majority of childhood cancer survivors do not receive recommended risk-based care [34]. The small country of Slovenia is, in this respect, in an advantageous position, with one pediatric center for treatment in close cooperation with the adult outpatient clinic at the Oncological Institute. The main advantage of the study presented here is that it is performed on a systematic database of the entire population of the children cancer patients in Slovenia [10] that have been continuously followed-up in the same clinic under the unique opportunity for an » ongoing communication between the cancer center that provided acute care for the patient and the healthcare facility providing follow-up care »  [35].

The results of the Kaplan-Mayer analysis show that the incidence of late sequelae in childhood cancer patients five and ten years after treatment in Slovenia steadily decreases from 75% in the group of patients diagnosed before 1975 to 55% in the patients diagnosed after 1995 (see Table 3). The results show that the impact of individual therapies on late sequelae incidence differs: we observe a high and significant impact of surgery on the incidence of late sequelae five and ten years after treatment, a lower but still significant impact of radiotherapy, and almost no impact of chemotherapy (see Figure 4).

The Cox regression analysis of late-sequelae risk reveals that three studied factors significantly influence the risk. The most important factor is the treatment modality. Combinations of treatments that include surgery significantly increase the risk for late sequelae: the estimated hazard ratio of 3.80 for the combination of surgery, radio-, and chemotherapy, 3.25 for the combination of surgery and radiotherapy, and 2.49 for surgery and chemotherapy (see Table 4). Furthermore, age at diagnosis also significantly influences the hazard ratio for late sequelae from 1 for the reference group of patients diagnosed when below 5 years of age to significantly lower ratios of 0.65 and 0.40 for the patients diagnosed between 5 and 10 years as well as 10 and 16 years of age, respectively (see Table 4). Finally, the primary diagnosis also significantly influences the risk for late sequelae, with Leukemia leading to significantly lower late-sequelae risk as compared to all other diagnoses and renal tumors leading to the highest hazard ratio (see Table 4). However, deeper analysis of these results (see Table 5) shows that this is mostly due to the selection of the appropriate treatment modality for the specific primary cancer diagnosis.

In this paper, we focus on the influence of individual risk factors on incidence of and risk for late sequelae other than none. Two directions for immediate further work are evident. First, survival analysis can be used to explore the incidence of and risk for more serious (mild, moderate, or severe) somatic sequelae. Second, survival analysis can be combined with multivariate statistical methods to further explore the interactions among different risk factors with respect to their joint influence on the late sequelae of childhood cancer treatment.

## Competing interests

The authors declare that they have no competing interests.

## Authors’ contributions

NE participated in preparation of the data, preliminary statistical analysis, and the design of the study. LT participated in the design of the study, performed the statistical analysis, and wrote the manuscript. BJ conceived the study, participated in its design and coordination, and helped with drafting the manuscript. All authors have read and approved the final manuscript.

## Ethical approval

The research on humans performed in the paper is in compliance with the Helsinki Declaration and approved by the National Medical Ethics Committee of the Republic of Slovenia.

## Supplementary Material

Additional file 1 Late somatic sequelae classification: Detailed specification of the late somatic sequelae classification procedure. Click here for file
