# Supplementary material for: Late somatic sequelae after treatment of childhood cancer in Slovenia
Source: BMC Res Notes. 2012 May 24;5:254. doi: 10.1186/1756-0500-5-254 (PMC3444361; doi:10.1186/1756-0500-5-254)
Supplement: Additional file 1 — Late somatic sequelae classification: Detailed specification of the late somatic sequelae classification procedure. [file 1756-0500-5-254-S1.doc]

## Appendix: Late Somatic Sequelae Classification

### Mild (Score 1)

- post-irradiation tissue atrophy
- post- op. tissue deficit
- alopecia
- microcephaly
- growth deficit
- splenectomy
- nephrectomy
- unilateral orchiectomy
- unilateral ovariectomy
- unilateral thyroid lobectomy
- limb-perserving surgery
- one endocrinological deficit requiring substitution
- abnormality with laboratory test of one organ with mild clinical symptoms (dispnea, tachycardia, hypertension)
- gastrointestinal symptoms (pain, bowel movements)
- hearing loss (mild)
- mild loss of vision (keratitis catharact)
- hyperpigmentation (numerous nevi)
- mild neurological deficit (mild ataxia, nystagmus)
- obesity

### Moderate (Score 2)

- severe or facial post-irradiation tissue atrophy
- Soft tissue atrophy with aplasia of the breast
- Vaginal stenosis
- Uterus atrophy
- Painful scoliosis
- Any two mild sequelae (nephrectomy & scoliosis, hypertension, growth deficit, splenectomy & endocrinological deficit)
- Two or more positive laboratory test & moderate clinical symptoms (thyroid,heart, lung, kidney, ginecomastia)
- Limb-perserving surgery & complications ( reoperation, osteomyelitis)
- Limb-perserving surgery & two or more positive laboratory tests and clinical symptoms
- Above knee amputation
- Total thyreoidectomy for primary carcinoma
- Mastectomy for primary carcinoma
- Azoospermia
- Panhypopituitarism
- Loss of one eye
- Chronic meningitis
- Orchidectomy& radical lymph node dissection
- Not life treatening second malignancy, (skin carcinoma)
- Moderate neurological deficit (hemiparesis)
- Epilepsy (responding to mediction)

### Severe (Score 3)

- Two moderate ( eye enucleation & sever post irradiation facial atrophy)
- Panhypopituitarism & other laboratory tests with symptoms (diabetes, heart, epilepsy)
- Complete loss of hearing
- Blindness
- Limb exarticulation
- Hemipelvectomy
- Sterility, women
- Complete loss of testicular function
- Cystostoma
- Dwarfism
- Severe disabling neurological deficit (paraplegia)
- Epilepsy, not responding to medication
- Any two or more moderate sequelae (hemiparesis & epilepsy)
- Severe one organ faillure (heart, lung, kidney)
- Two or more moderate organ failures (heart & lung & kidney)
- Second malignancy, life threatening or requiring mutilating surgery (brain tumor, thyroid ccarcinoma)

### Death (Score 4)

- Due to late sequelae

### Notes on the Classification

This classification presented her is based primarily on the nature of the sequelae and only secondary on their degree. Obviously, severe degrees of »light« or »moderate« sequelae should move them to a higher category. All according to the judgement of the examining doctor. While »mild sequelae« might be easy to classify, it is often difficult to classify moderate and severe sequelae, when several are combined. Tissue atrophy and hypothyreosis is a quite common combination in patients treated for HD, classified as moderate sequelae. Combinations of moderate sequelae classified as severe, might be sometimes very difficult, as this depends so much on the individuality of the patient. Futhermore, there may be combinations of severe sequelae, e.g., sterility and secondary tumor, that are only classified as severe and not as a special category.

Note however, that the difficulties in distinguishing between categories do not influence the results of the Kaplan-Meier and Cox regression analysis, since the event considered there is the incidence of any late sequelae with a score above 0, i.e., the incidence of late somatic sequelae of any category listed in this Appendix.
